# Can species adapt to drought using multiple strategies? Lessons from the California poppy

**DOI:** 10.1111/nph.71105

**Published:** 2026-04-02

**Authors:** Stuart T. Schwab, Kristal Lam, Finn Thornton, Rachel Brown, Joe Kesler, Cory Merow, Jason P. Sexton, Elsa E. Cleland

**Affiliations:** ^1^ Department of Ecology, Behavior, and Evolution University of California San Diego La Jolla CA 92093 USA; ^2^ Department of Biology Stanford University Palo Alto CA 94305 USA; ^3^ Eversource Energy Center and Department of Ecology and Evolutionary Biology University of Connecticut Storrs CT 06269 USA; ^4^ Department of Life and Environmental Sciences University of California Merced Merced CA 95343 USA

**Keywords:** eco‐physiology, *Eschscholzia californica*, functional traits, glasshouse experiment, plasticity, population

## Abstract

- Plants can escape drought by completing life cycles early, tolerate drought by increasing physiological limits, or avoid drought stress by obtaining or using water more efficiently. It remains unclear whether strategies vary within species across their distributional ranges due to trade‐offs, and whether species can exhibit plasticity in multiple traits simultaneously.
- We grew 19 populations of *Eschscholzia californica* collected along an aridity gradient in a glasshouse with high or low water, then applied a terminal drought treatment, and measured the responses of growth and functional traits.
- We found clinal variation in drought adaptation strategies; populations from arid sites exhibited escape phenotypes, while populations from mesic areas exhibited avoidance phenotypes. In response to low water, plants displayed plasticity in traits associated with both avoidance and tolerance strategies, and this plasticity was expressed consistently across populations. By contrast, specific root length (SRL) displayed clinal variation in plasticity; more arid sites had higher SRL (longer/thinner roots), and SRL increased the most in response to low water in the populations from arid sites.
- Our experiment demonstrates that frameworks developed to predict interspecific variation in drought adaptation strategies can also operate intraspecifically, with implications for wildflower conservation in the face of increasingly frequent droughts.

## Introduction

Climate change is a major driver of biodiversity loss world‐wide (Hald‐Mortensen, 2023); where species must withstand the changes, adapt in place, or move to areas with newly suitable conditions (Thurman *et al*., 2020). Species with ranges that span climatic gradients can have populations adapted to local conditions (Leimu & Fischer, 2008), due to heritable, continuous (clinal) trait variation across their range that is associated with climate (De Frenne *et al*., 2013; Halbritter *et al*., 2018), and varying responses to climatic factors across populations (Krushelnycky *et al*., 2020). Projections of future species responses to climate change that assume consistent responses across species ranges can differ greatly from approaches that incorporate clinal variation (e.g. Benito Garzón *et al*., 2011; Valladares *et al*., 2014; DeMarche *et al*., 2019).

While climate change has many aspects, here we focus on rising aridity, which is already causing declines in plant diversity (Harrison *et al*., 2015; Li *et al*., 2019). Rising temperatures in recent decades have resulted in global increases in aridity and drought, and declines in soil moisture (Dai, 2012). Global tree‐ring datasets show that plant growth is becoming increasingly water‐limited, corresponding with this increase in aridity (Babst *et al*., 2019). Furthermore, aridity is projected to increase in the coming century, even in areas where precipitation is expected to rise, due to increased evaporative demand with higher temperatures (Scheff & Frierson, 2015; Berg *et al*., 2016; Gebrechorkos *et al*., 2025). This makes adaptation to increased aridity one of the most globally relevant mechanisms modulating plant responses to climate change. Hence, quantifying clinal variation in drought adaptation strategies can inform species and population‐level vulnerability to rising aridity, a critical aspect of climate change for plants. In woody plants, drought strategies can be described as isohydric, where plants prioritize maintaining water status at the cost of carbon starvation, or anisohydric, where plants maintain photosynthesis at a low level at a higher water cost (McDowell *et al*., 2008); however, nonwoody plants are constrained in their hydraulic tolerances (Skelton *et al*., 2017), and carbon storage (Schärer *et al*., 2023).

Nonwoody plants' drought strategies must rely more heavily on morphological means of drought adaptation (Lambers *et al*., 2008), where three broad categories of drought adaptation strategies have been proposed to explain herbaceous plant responses to drought: escape, avoidance, or tolerance (Ludlow & Muchow, 1990; Kooyers, 2015). Drought escape is associated with fast growth and often an annual life‐history strategy, whereby plants escape the physiological stress of drought by being active only at times when soil moisture is plentiful. Highly variable precipitation regimes as well as higher temperatures select for annual life‐history strategies (Boyko *et al*., 2023), in addition to distinct rainy seasons which necessitate early flowering before the onset of seasonal drought (Takeno, 2016). The escape strategy also necessitates fast growth to complete their life cycle in a short growing season, which requires the capacity for high‐resource acquisition, through high specific root length (SRL) and high specific leaf area (SLA; Wright *et al*., 2004; Reich, 2014). By contrast, drought avoidance strategies for nonwoody plants limit water loss via phenotypes, such as highly dissected or lobed leaves (Talbert & Holch, 1957), which can reduce leaf temperatures (Gurevitch, 1988; Schuepp, 1993). Plants with drought avoidance strategies also have lower allocation to aboveground tissues exposed to water loss, thus increasing their root mass fraction (RMF, Eziz *et al*., 2017), and allowing them to access deep soil moisture. Drought avoidance strategies are most likely to occur with mild droughts during growing seasons or with consistent but low water availability (McDowell *et al*., 2018). Finally, plants can tolerate droughts through physiological mechanisms like upregulating osmoprotectants or increasing secondary compounds to lower turgor loss point (TLP; Wang *et al*., 2022, Cao *et al*., 2023), a strategy most likely to evolve in response to short‐term, intense droughts (Kooyers, 2015; McDowell *et al*., 2018).

Theoretical frameworks associated with escape, avoidance, and tolerance were developed to understand how drought adaptation strategies vary among species, but it is less clear whether intraspecific trait variation follows similar patterns. Theories regarding plant growth strategies are often structured around a trade‐off between species that grow quickly and dominate under high‐resource conditions, and species that can persist under low‐resource conditions by having trait values associated with high‐resource‐use efficiency (e.g. Grime, 1977; Wright *et al*., 2004). However, across environmental gradients, trait coordination predicted by this trade‐off may be stronger across than within species (Cornwell & Ackerly, 2009; Angert *et al*., 2014; Anderegg *et al*., 2018). Within woody species, natural selection experiments have demonstrated that drier microclimates may accelerate phenology and increase SLA, while reducing water use efficiency (Blanco‐Sánchez *et al*., 2022), thereby selecting for an escape response. Conversely, investigations of clinal variation in trees have demonstrated populations in wetter areas have higher growth, higher gas exchange, and lower sapwood capacitance following an acquisitive–conservative axis of growth and resource use (Ramírez‐Valiente *et al*., 2025). Within herbaceous plants, a recent experiment found that smaller plants with more conservative resource trait phenotypes survived longer in drought (Funk *et al*., 2024); however, reproduction during drought is a key evolutionary component of drought responses where a trade‐off between survivorship during drought and reproduction may alter the selection outcomes of drought responses (Lauder *et al*., 2019). Hence, it is unclear how trade‐offs among drought strategies might structure clinal variation across ranges that span aridity gradients in herbaceous plants.

In addition to the suites of coordinated traits associated with drought adaptation, species may exhibit plasticity in key functional traits. For instance, plants experiencing drought can respond plastically by increasing RMF (Eziz *et al*., 2017; Westerband *et al*., 2019), reducing SLA, increasing leaf dry matter content, increasing water use efficiency (Blumenthal *et al*., 2021), and accelerating phenology (Gugger *et al*., 2015; van Dyke & Kraft, 2025), although this phenology response might be limited to early‐season species (Castillioni *et al*., 2022). Across a species' range plants may exhibit consistent plasticity, have clinal variation in their plasticity (Nagy *et al*., 2024), or display different patterns depending on the trait (McLean *et al*., 2014). In some cases, plasticity can be a larger driver of intraspecific functional trait variation than population‐level differences (e.g. Ren *et al*., 2020; Kerr *et al*., 2022). As of yet, it is unclear whether plants with different drought adaptation strategies can display similar plasticity in key traits, or whether physiological trade‐offs constrain plasticity in species traits and drought adaptation strategies (Volaire, 2018).

California poppy, *Eschscholzia californica*, is an exceptional model system to test how clinal variation influences drought adaptation strategies, and to quantify plasticity in drought response traits across a species range. *E. californica* occupies a Mediterranean climate with cool wet winters and dry summers and experiences a dry season throughout its range which spans from Baja California, Mexico, to Washington state, United States. This species is an obligate outcrosser with minimal selfing, and low viability in the rare cases of self‐pollinated individuals (Cook, 1962). The southern portion of the range has both lower average rainfall and greater interannual rainfall variation (Dettinger *et al*., 2011), and the entire region is expected to experience increasingly severe droughts in the future (Mann & Gleick, 2015; Douville *et al*., 2021). *Eschscholzia californica* exhibits large intraspecific variation and at one point was described as up to 90 distinct species or sub‐species (Still, 2021). In particular, *E. californica* displays life‐history variation across its range, with perennial populations in northern, more mesic areas, and annual populations with greater potential for seed dormancy in the southern more arid region (Cook, 1962). More mesic populations also tend to have greater belowground allocation (Boucher, 1985; Ryan & Cleland, 2021) and more conservative resource acquisition traits (Leger & Rice, 2007). Recent work found that southern, arid populations exhibit greater fitness when exposed to water stress compared with more northern populations (Ryan & Cleland, 2021). Hence, we expected to find similar variation in fitness in response to our experimental treatments, as well as variation in drought adaptation strategies across the range of *E. californica*, specifically associated with clinal variation in key fitness‐related traits. We additionally sought to understand whether plasticity varied along the cline (i.e. clinal variation in plasticity), and whether plants were able to exhibit plasticity in multiple traits simultaneously.

Clinal variation is often quantified by growing populations together in common gardens (Lortie & Hierro, 2022). When coupled with experimentally controlled environmental manipulations, common gardens are ideal for elucidating the degree to which intraspecific variation is driven by clinal variation vs plastic responses to the environment (Etterson *et al*., 2016). This approach can also reveal how populations vary in functional traits and fitness, and the expected shifts in functional traits and fitness due to contemporary evolution in the face of future climate change (Lucas *et al*., 2024). Using *E. californica* as a model system, and employing a common garden approach along with experimental watering and terminal drought treatments, we asked (1) Is there clinal variation in functional traits associated with drought response strategies? and (2) Does trait plasticity in response to low water availability and to a terminal drought mirror the clinal patterns? Based on previous research (Cook, 1962; Ryan & Cleland, 2021), we expected to find that southern, arid populations would exhibit a drought escape phenotype (i.e. high SRL, high SLA, and fast time to flower), whereas northern, mesic populations would exhibit a drought avoidant phenotype with corresponding suites of functional trait phenotypes. Finally, based on fundamental theory we expected to find greater plasticity in traits associated with drought adaptation strategies in the southern, arid populations that experience greater interannual variation in rainfall (Schlichting, 1986). These differences in plasticity for arid sites have also been experimentally tested in other species, (Heschel *et al*., 2004), further suggesting that plant populations in more arid sites may have greater plasticity in key drought adaptation traits.

## Materials and Methods

### Seed collection and preparation for experiment

**Fig. 1:**
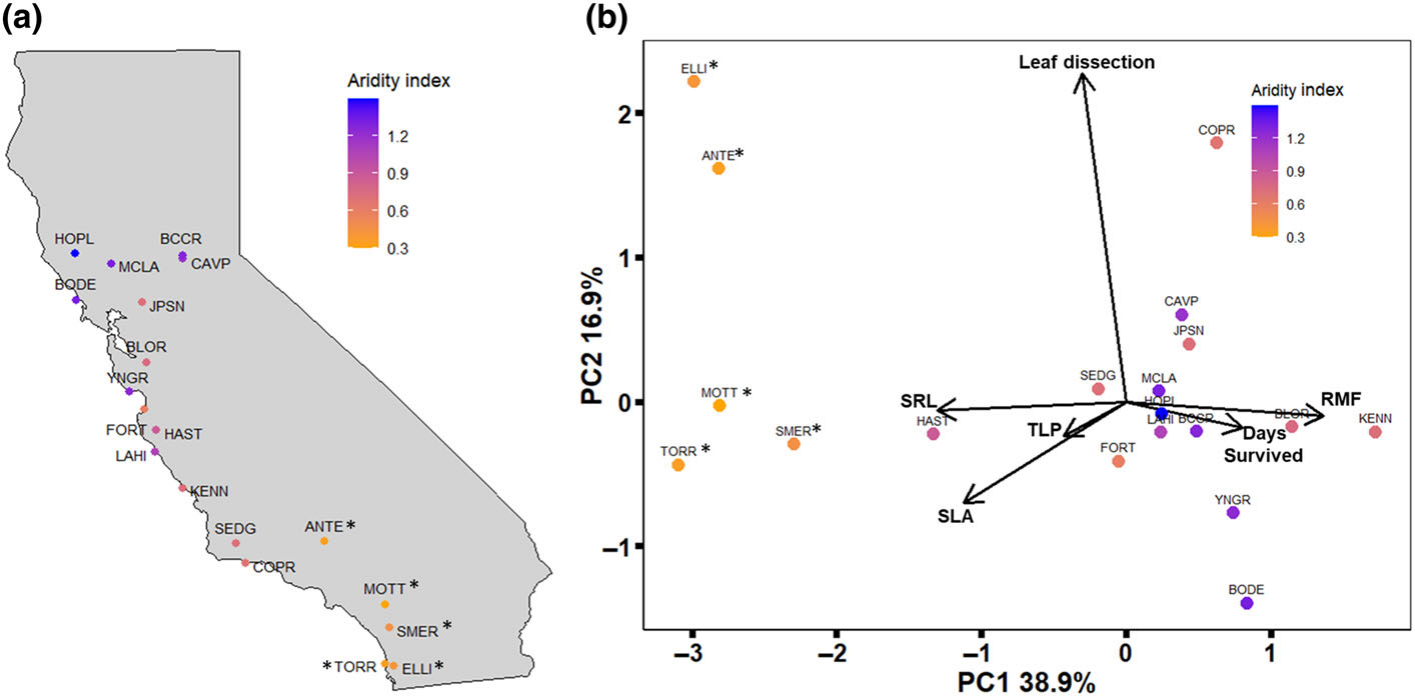
Source *Eschscholzia californica* population locations across the state of California (a) and multivariate trait space of selected populations visualized via principal components analysis (b). Colors indicate the aridity of each location (see Materials and Methods section for details on calculation of the aridity index, and Supporting Information Table S1 for complete environmental data for each collection site and associated population). Asterisks indicate populations that demonstrate an annual life habit based on demographic surveys (Brown, 2025), other field observations, and common garden experiments. In (b), each point is the population average for traits measured in the continued watering treatments (averaged across the high and low watering regimes), except for ‘days survived’, which was only measured in the terminal drought treatment. The percentages on each axis in (b) are the amount of variation explained by the traits. RMF, root mass fraction; SLA, specific leaf area; SRL, specific root length; TLP, turgor loss poin.

We collected seeds from wild *Eschscholzia californica* Cham. populations in May through June of 2023 (Fig. 1, see Supporting Information Fig. S1 for covariance with other environmental variables, see Table S1 for population averages for each environmental covariate, environmental data downloaded from PRISM Climate Group) from 20 to 30 individual maternal plants, with a minimum spacing of 5 m where possible to represent local environmental variation. Before planting, we surface sterilized seeds with a 5000 ppm bleach solution, and then, after rinsing with deionized water twice, we added a 500 ppm gibberellic acid solution overnight to break dormancy and increase germination rate (Fox *et al*., 1995). The concentration of gibberellic acid that we utilized does not significantly alter flowering time (unpublished data), and additional pilot measurements indicate no influence on germination timing (data not shown). We planted seeds the day following gibberellic acid treatment.

We chose seven maternal lines from each population to plant into 5‐l tree pots (Stuewe & Sons CP512) filled with locally sourced topsoil (Greatsoil LLC: San Marcos, CA, USA). Soil microbes can influence local adaptation in plants (Petipas *et al*., 2021), including their responses to drought (e.g. Lau & Lennon, 2012). However, a prior study which compared drought responses of *E. californica* populations with and without soil inoculations from their collection sites found that matching soil provenance increased plant biomass only 4% and did not influence fitness responses to drought (Ryan & Cleland, 2021). The number of maternal lines we could include from each of the 19 populations was constrained by the number of treatments, and total number of pots we could fit in the glasshouse. Three seeds per pot were added 1 cm below the soil surface on 18 January 2024. No seedlings emerged within the first week, where after an additional 2 wk (i.e. 3 wk total), we added another three seeds for pots that did not germinate in the first round. Two weeks after the second seeding (i.e. 5 wk since first seeding, 2 wk after first cohort emergence), we transplanted within maternal lines from pots that had more than one germinant to pots with no seedlings and weeded down to one seedling per pot in all pots. Seedlings were placed in cohorts based on which week they emerged. All plants received an intermediate amount of water for the first 5 wk to facilitate seedling establishment.

### Glasshouse experimental design

**Fig. 2:**
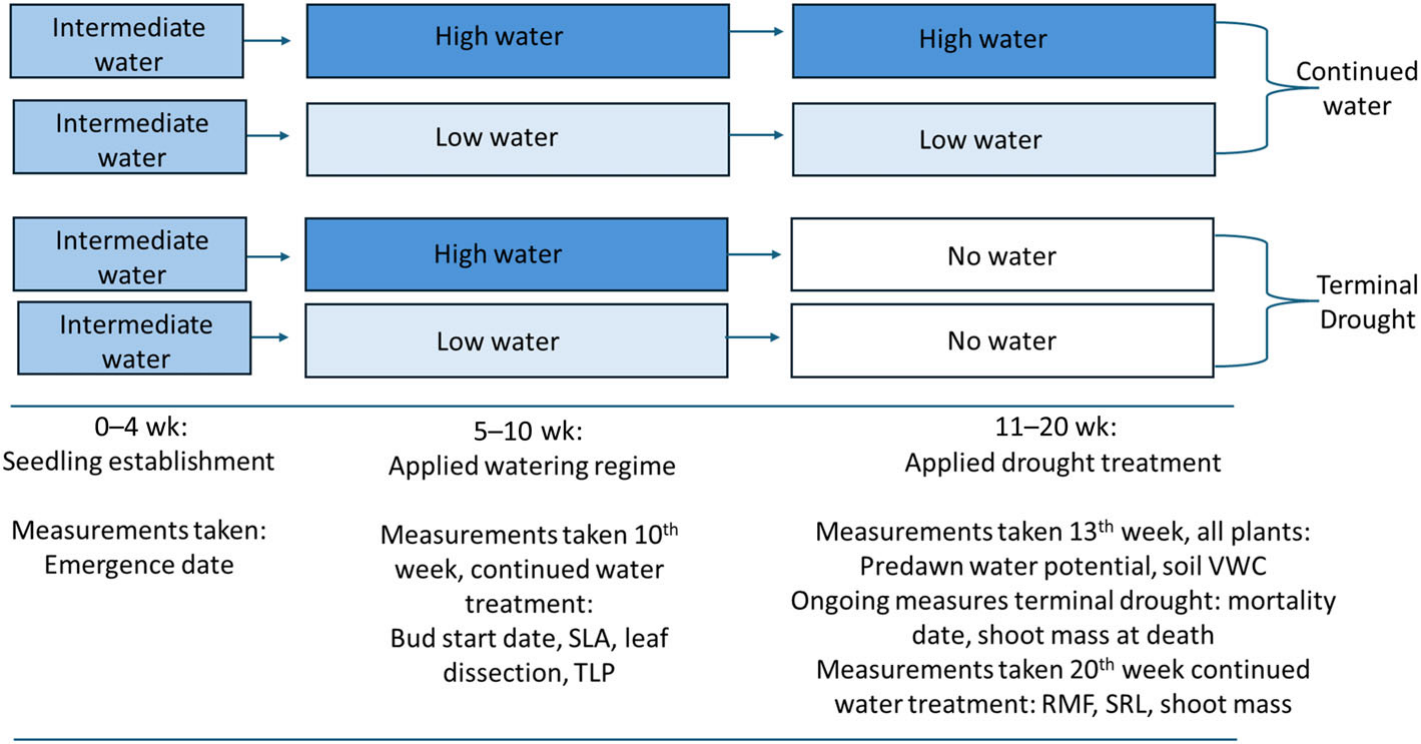
Timeline of watering treatments and measurements of plant response variables for *Eshscholzia californica*. Measures that were taken before implementing terminal drought, but only in the continued water treatment, include specific leaf area (SLA cm^2^ g^−1^), leaf dissection index (cm^−1^), and turgor loss point (TLP, MPa). After terminal drought was applied for 3 wk, predawn water potentials were taken on all plants (water potential, MPa), and mortality surveys were performed daily. Shoot and root biomass, specific root length (SRL cm g^−1^), and root mass fraction (RMF) were measured during a final destructive harvest, where only continued watering treatments were analyzed for both root traits. Plants in the continued watering treatment were harvested in the 20^th^ week postemergence, and plants in the terminal drought were harvested the day of death.

We performed a factorial design for the glasshouse experiment (Fig. 2), where plants were assigned high or low watering treatment for 5 wk after initial establishment (i.e. 10 wk total); then, they were either subjected to a terminal drought (no water added) or they continued to receive water for an additional 10 wk (i.e. 20 wk from emergence to harvest). We used a split block design for the high and low watering treatments, which were designed to mimic the field conditions. The high watering regime mimicked the most mesic population (Hopland Reserve, 80 ml per pot every other day), which saturated the soil, and the low watering regime mimicked the most arid population's climatic conditions (Motte Rimrock Reserve, 30 ml per pot every 4 d) which did not fully saturate the soil. The experiment thus had a planned total of 532 plants (19 populations × 7 maternal lines × 2 watering treatments (i.e. high or low) × 2 drought treatments (i.e. continued watering and terminal drought; Fig. 2). However, several maternal lines did not have enough germination to be represented in all treatments, and 14 of 133 lines did not emerge at all resulting in a total of 405 plants (see Table S2 for sum of plants per maternal line and population).

### Measurements

We downloaded local weather station data from a local National Oceanic and Atmospheric Administration Center for Environmental Information (station: La Jolla 2.2 NE, CA US) to acquire ambient daily temperature averages for the length of the experiment. We also placed three temperature sensors (Analog Devices®) evenly throughout the glasshouse starting the week of inducing watering regimes (i.e. high or low water), and air temperature was measured every hour. We also estimated photosynthetically active radiation (PAR) with an Accupar LP‐80 light wand in five locations outside of the glasshouse and five locations inside of the glasshouse at noon over five nonconsecutive cloudless days.

We conducted daily emergence surveys for 3 wk after planting for both rounds of planting. We also performed daily budding surveys beginning 10 wk after initial emergence to determine when plants began reproductive allocation, and we performed daily mortality surveys starting 14 wk after initial emergence (i.e. 4 wk into terminal drought). We determined mortality as complete aboveground dieback via visual and mechanical assessment (i.e. 100% yellow curled‐in leaves where the center whorl crumbles at light touch).

One week before implementing the terminal drought treatment (i.e. 10 wk postemergence) we removed the two most recently fully formed leaves from the continued water subset of high and low watering to measure TLP via vapor pressure osmometry (EliTech VAPRO 5600: Logan, UT, USA) as well as SLA and leaf dissection index. We submerged leaves for TLP in deionized water overnight before osmometry, and calculated TLP following Bartlett *et al*. (2012). For SLA, we scanned fresh leaves flattened with small pieces of tape, and the area and perimeter were measured using ImageJ (Schneider *et al*., 2012). We calculated leaf dissection as $\frac{\text{leaf perimeter}\;\left(\mathrm{cm}\right)}{\;\text{leaf area}\;\left(cm^{2}\right)}$ where higher values are more dissected, and SLA was calculated as $\frac{\text{leaf area}\;\left(cm^{2}\right)}{\text{leaf}\;\mathrm{dry}\;\text{mass}\;\left(g\right)}$ where higher values are larger, thinner, less dense leaves. We removed three outlier values for SLA where, upon later inspection, small pieces of leaves had stuck to the tape causing measurement error.

After 3 wk of simulated drought (i.e. 13 wk postemergence), we measured soil volumetric water content (VWC) with a Field Scout TDR 100 Soil Moisture Sensor, and predawn water potentials (a measure of how the treatments impacted water availability to plants) the following night with a Scholander pressure chamber (PMS Instrument Co. Model 1000 Pressure Chamber). We sampled predawn water potential measures at least 2 h after sunset and completed all measures before sunrise, by removing the first fully formed leaf with a razorblade and immediately placing the leaf in the pressure chamber.

We harvested plants in the terminal drought treatment on the day of complete dieback, and harvested all plants in the continued water treatment 20 wk postemergence (i.e. 10 wk into comparison with imposed drought). We passed all soil from pots in the continued water treatment over a 4 × 4 mm metal mesh to collect roots of the living plants. We then placed roots in a wet paper towel before scanning (Epson Perfection V600 Photo scanner: Seiko Epson Corporation, Suwa, Nagano, Japan) in a thin film of water. We then used the U‐Net semantic segmentation network from the RootDetector program (Peters *et al*., 2023) to automatically detect the roots in the image scans, skeletonized the images with RootDetector (Gillert *et al*., 2023), and then measured root length with the Rhizovision software (Seethepalli & York, 2020). We then calculated SRL as $\frac{\text{root length}\;\left(\mathrm{cm}\right)}{\text{root mass}\;\left(g\right)}$. We dried shoots and roots in a 40°C oven for 5 d before weighing for dried biomass. Shoot mass is our proxy for plant fitness (Younginger *et al*., 2017).

### Analysis

We calculated the aridity index for each location based on Thornthwaite (1948), defined as the ratio of precipitation to potential evapotranspiration, where greater values indicate more mesic areas. In this ratio, aridity values less than one indicate that evapotranspirative demand is greater than precipitation, and values greater than one indicate precipitation is greater than evapotranspirative demand. The range of aridity investigated spans from 0.29 at the most arid site to 1.32 at the most mesic site (see Table S1 for site averages; Fig. S1 for correlations with other environmental covariates, environmental data downloaded from PRISM Climate Group).

We used the Thornthwaite function in the Spei R package (Beguería & Vicente‐Serrano, 2023) to calculate potential evapotranspiration. To assess multivariate trait space, we performed a principal components analysis (PCA) using the vegan package (Oksanen *et al*., 2025). For all linear models, we compared linear fits with quadratic fits via Akaike Index Criterion (AIC) and Shapiro–Wilk test for normality of residual distribution for all models to determine the best fit and necessary transformations (Table S3). We analyzed soil VWC, predawn water potential and shoot mass with quadratic mixed models, assuming a Gaussian distribution for the residual error and as one family for Benjamini–Hochberg corrections (Benjamini & Hochberg, 1995). For these models, aridity index of the home population, watering regime (i.e. high or low water) and drought treatment (i.e. continued watering or terminal drought) were the fixed effects with cohort and population as random terms. We estimated the variance explained by fixed effects compared with the random terms (i.e. population and cohort) with the MuMIn package (Bartoń, 2025).

Our focal traits are associated with three drought adaptation strategies in nonwoody plants: escape (fewer days emergence to flower, and resource acquisitive phenotypes: high SLA and SRL), avoidance (morphological traits associated with retaining water or reducing water loss: high RMF and highly dissected leaves), and tolerance (phenotypes associated with hydraulic tolerance (i.e. low TLP and more days survived in drought; Ludlow & Muchow, 1990, Kooyers, 2015)). We measured these traits on plants in the continued watering treatments (both high and low watering regimes), except for days survived in drought, which was only measured on plants in the terminal drought treatment. A large portion of the populations did not flower under the glasshouse conditions (99/210 plants in continued water, 45/195 plants in terminal drought), and the number of individuals flowering was not evenly distributed across the range (Fig. S2). Therefore, we calculated the probability of flowering per individual as a binomial mixed model and performed correlations with other traits associated with resource acquisition (i.e. days emergence to flowering, SLA, and SRL; Fig. S3).

For our analysis of drought adaptation strategies, we used aridity index of the home population, watering regime (i.e. high or low water) and their interaction as fixed effects with quadratic mixed models, assuming a Gaussian distribution for the residual error, with source population and cohort as random terms except for the probability of flowering, which had a binomial distribution. We assessed model fits by both Shapiro–Wilk test for normality of model residuals, as well as a visual inspection using the qqnorm function (R Core Team, 2023). We performed natural log transformations on SLA, SRL, leaf dissection, days alive in drought, TLP, and VWC based on model selection, while shoot mass and RMF underwent square root transformations (Bolker, 2008). All trait focused models underwent FDR Benjamini–Hochberg corrections as a second family (Benjamini & Hochberg, 1995). We estimated the variance explained by fixed effects compared with the random terms (i.e. population and cohort) with the MuMIn package (Bartoń, 2025). Untransformed data are presented in all figures, with corresponding figures containing transformed data displayed in Figs S4 and S5.

We compared linear vs quadratic fits with orthogonal polynomials using the poly(x,2) function in lme, and selected models based on AIC scores (Table S2). All linear and quadratic models were performed in R (v.4.3.3) using the lme() function from the nlme package (Pinheiro & Bates, 2000), specifying type‐II tests using the ANOVA function in the car package (Fox & Weisberg, 2019) for our binomial model, and marginal means via Wald *F* test for our linear models. We performed *post hoc* Tukey Honestly Significant Difference (HSD) comparisons with the emmeans() function from the emmeans package with a false discovery rate correction via Benjamini‐Hochberg (Lenth, 2024). We used the cor.test function (base R) to test correlations between probability of flowering and other resource acquisitive and phenological traits. Full summary statistics for VWC, predawn water potential, and growth responses are in Table 1, and trait phenotype responses are in Table 2.

**Table 1:** Summary statistics for top selected models of soil volumetric water content, water stress (predawn water potential), and *Eschscholzia californica* plant fitness (shoot mass), in response to site aridity, the experimental watering regime, and the experimentally imposed terminal drought.

	VWC		Predawn WP		Shoot mass	
	*F*	*P*	*F*	*P*	*F*	*P*
Aridity index (quadratic)	0.11	0.949	1.13	0.517	0.62	0.758
Watering treatment	368.41	**< 0.0001**	19.98	**< 0.0001**	51.01	**< 0.0001**
Drought treatment	62.80	**< 0.0001**	63.52	**< 0.0001**	78.54	**< 0.0001**
Aridity index × Watering treatment	0.25	0.932	0.93	0.591	1.93	0.252
Aridity index × Drought treatment	2.09	0.231	0.29	0.931	0.10	0.948
Watering × Drought treatment	87.96	**< 0.0001**	9.58	**0.005**	9.63	**0.005**
Aridity × Watering × Drought	0.00	0.100	0.33	0.932	0.16	0.948
Variance explained by fixed effects	64.3%		33.2%		40.3%	
Variance explained by random effects	6.3%		13.5%		10.8%	

These models include both terminal drought and continued water treatments, and therefore have a three‐way interaction between watering regime, drought treatment, and aridity index of source population. Each column represents an individual response variable, and the rows are fixed effects and their interactions where source population and cohort are random terms. The first number is the *F* ratio, and the second number is the *P* value where significant effects are bold. The aridity index is shortened to aridity, watering treatment is shortened to watering, and drought treatment is shortened to drought for all interactions due to space constraints in the table. The final two rows are the variance explained by all fixed effects for each model, and the variance explained by the random terms (i.e. cohort and population).

**Table 2:** Summary statistics for top selected models of *Eschscholzia californica* traits relating to drought response strategies, in response to site aridity and the experimental watering regime.

		Probability of flowering		SLA		SRL	
		χ^2^	*P*	*F*	*P*	*F*	*P*
Escape	Aridity index	17.99	**0.0001**	18.14	**< 0.0001**	17.72	**< 0.0001**
Escape	Watering treatment	0.00	0.997	1.98	0.198	4.6	**0.050**
Escape	Aridity × Watering	1.19	0.323	0.45	0.719	4.39	**0.023**
	Variance fixed effects	61.6%		28.8%		29.4%	
	Variance random effects	18.7%		13.0%		28.1%	

		RMF		Dissection index	
		*F*	*P*	*F*	*P*
Avoidance	Aridity index	33.22	**< 0.0001**	4.81	**0.017**
Avoidance	Watering treatment	13.80	**0.0005**	4.86	**0.046**
Avoidance	Aridity × Watering	1.88	0.198	0.24	0.817
	Variance fixed effects	50.3%		10.5%	
	Variance random effects	27.8%		17.9%	

		TLP		Days alive without water	
		*F*	*P*	*F*	*P*
Tolerance	Aridity index	2.98	0.077	9.06	**0.0004**
Tolerance	Watering treatment	17.99	**0.0001**	22.36	**< 0.0001**
Tolerance	Aridity × Watering	2.53	0.113	0.26	0.816
	Variance fixed effects	10.3%		22.4%	
	Variance random effects	29.0%		26.2%	

These models only include data from plants in the continued watering treatment, with the exception of days survived in drought, and thus these models only contain two‐way interactions between aridity index and watering regime. Each row represents an individual response variable, and each column is a fixed effect from the models, where source population and cohort are random terms. The final number is the *P* value and significant terms are in bold.

## Results

### Glasshouse conditions

The average ambient outdoor temperature for the duration of the experiment was 16.3°C; it was warmer inside of the glasshouse (average + 1.9°C). We found an average 27% reduction in PAR inside of the glasshouse at 1265 μmol m^−2^ s^−1^ compared with ambient outdoor levels which averaged 1465 μmol m^−2^ s^−1^ over five nonconsecutive full sun days.

### Multivariate assessment of functional traits

The PCA of our focal functional traits revealed variation among populations including a trade‐off between high‐resource acquisitive phenotypes with high SRL and SLA vs more conservative phenotypes with high RMF and longer survival in the terminal drought treatment on the first PC axis (Fig. 1b). Importantly TLP is negative, hence populations with higher SLA and SRL values would experience earlier turgor loss when encountering during drought stress. The average PC1 and PC2 scores for populations from more arid areas tend to load toward the resource acquisitive phenotype end of PC1, while the populations from more mesic areas tend to have average trait phenotypes on the conservative end of PC1. Leaf dissection index is orthogonal to the other trait phenotypes and loads on PC2.

### Water potential and biomass responses to low watering and terminal drought treatments

**Fig. 3:**
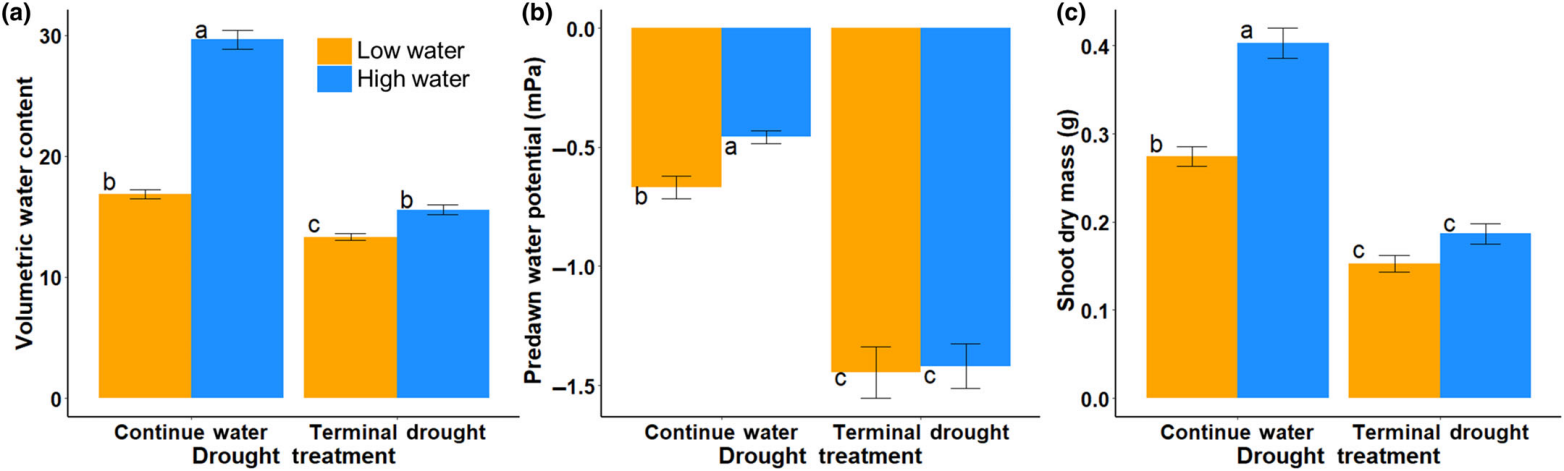
Volumetric water content (VWC) (a), predawn water potential (b), and shoot mass (c) of *Eschscholzia californica* in response to watering and terminal drought treatments. Color indicates high or low watering (orange = low water, blue = high water) with SE bars. Letters indicate results of Tukey honestly significant difference test. There was not clinal variation in VWC nor shoot mass; clinal variation in predawn water potential is shown in Supporting Information Fig. S1. See Table 1 for full summary statistics.

The treatments influenced soil moisture as expected (Fig. 3a; see Table 1 for summary statistics), as there was lower VWC in the low watering regime (*P* < 0.0001) and also in the terminal drought treatment compared with the continued watering treatments (*P* < 0.0001) 3 wk into the applied terminal drought. However, we found similar soil VWC between the continued low watering regime and the terminal drought high watering regime (Tukey HSD: *P* = 0.943) where all other comparisons between watering and drought soil VWC were as expected (drought treatment × watering regime *P* < 0.0001, Tukey HSD: low water × continued vs high water × continued *P* < 0.0001, low water × continued vs low water × drought *P* = 0.001, high water × continued vs low water × drought *P* < 0.0001, high water × continued vs high water × drought *P* < 0.0001, low water × drought vs high water × drought *P* = 0.011. Fig. 3a).

Consistent with these treatment effects on soil moisture, plants in the low watering regime had lower predawn water potential than the high watering regime (watering regime  < 0.0001, Fig. 3b; see Table 1 for summary statistics), indicating greater drought stress. Plants in the terminal drought treatment also had lower predawn water potential compared with plants in the continued water treatment (drought treatment *P* < 0.0001), with an average reduction of 0.87 mPa or 254% under terminal drought. There was also a drought × watering regime interaction (*P* = 0.0005; Fig. 3b), whereby in the continued water treatment, plants receiving high water had higher water potential than plants receiving low water (Tukey HSD *P* = 0.016). However, in the terminal drought treatment both watering regimes had similarly low water potentials (Tukey HSD *P* = 0.999).

Both the low watering regime and the terminal drought treatment reduced shoot mass (watering regime *P* < 0.0001, drought treatment *P* < 0.0001, Fig. 3c; see Table 1 for summary statistics). Within the continued water treatment, the high watering regime had greater shoot mass than the low water (Tukey HSD *P* = 0.007), but within the terminal drought treatment, both watering regimes had similarly reduced shoot mass (Tukey HSD *P* = 0.850; drought × watering regime *P* = 0.005; Fig. 3c).

### Trait variation across populations, experimental responses, and associated drought adaptation strategies

**Fig. 4:**
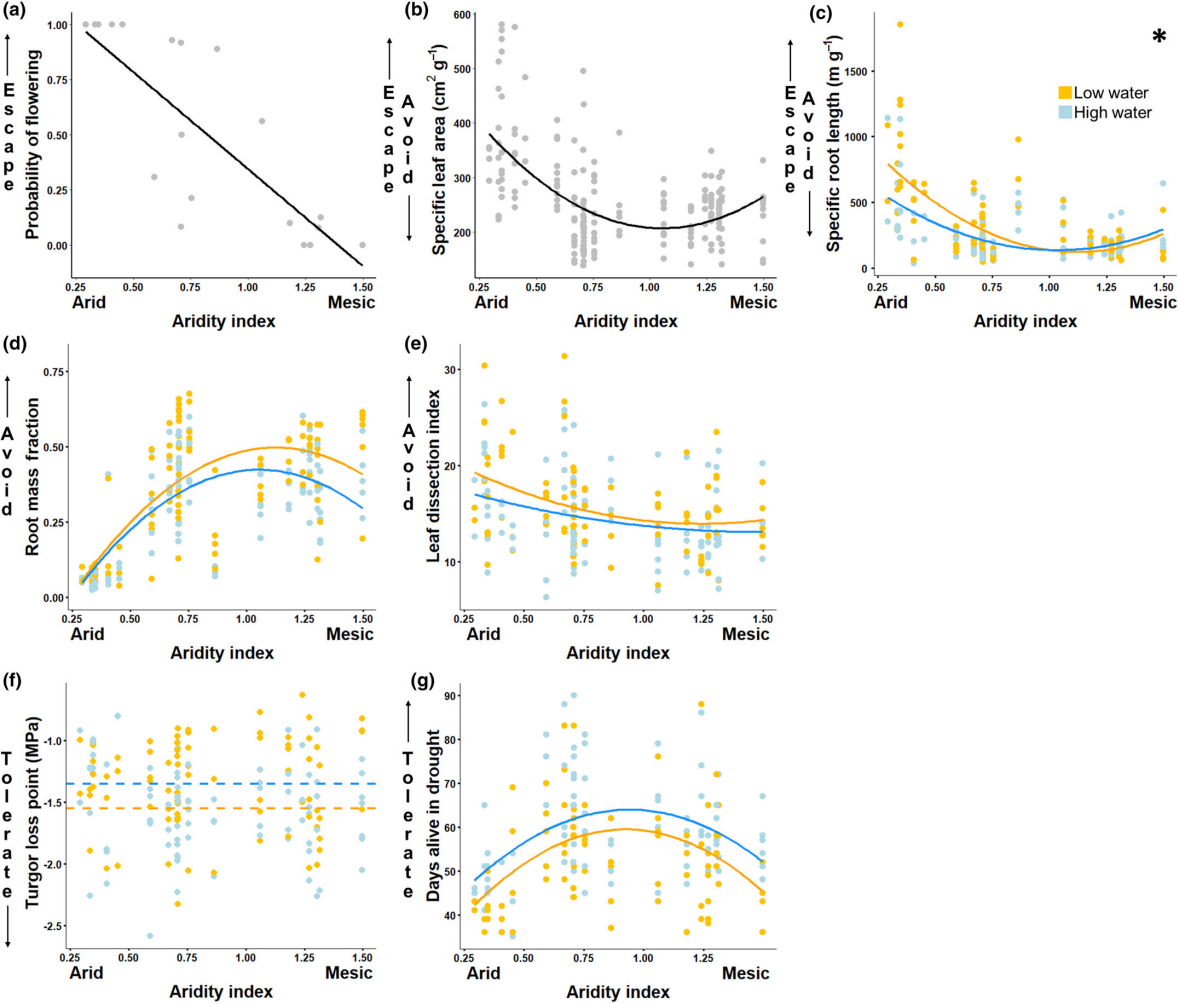
Clinal variation in functional trait phenotypes of *Eschscholzia californica*. Summary statistics for displayed relationships are in Table 2. Color indicates different watering treatments; orange is low water, blue is high water, and gray indicates there was no influence of watering treatment on the relationship. Lines indicate clinal relationships, where no lines indicate no detectable relationships, while dashed lines indicate watering regime differences but no detectable clinal relationships. The dashed color lines indicate that clinal variation did not have a detectable impact, but watering regimes were significantly different (Table 2). (a) Probability of flowering for each population. (b) Specific leaf area (SLA (cm^2^ g^−1^)). (c) Specific root length (SRL (m g^−1^)). (d) Root mass fraction (RMF). (e) Leaf dissection index (cm^−1^). (f) Turgor loss point (TLP, Mpa). (g) Days survived in terminal drought treatment.

*Escape:* the probability of flowering was greater in populations from more arid areas (aridity index *P* = 0.0001), independent of watering regime (*P* = 0.997, see Table 2 for summary statistics). Plants from more arid populations had higher SLA than plants from more mesic populations, with the lowest SLA in intermediate populations (aridity index *P* < 0.0001; Fig. 4b; see Table 2 for summary statistics). Similar to SLA, SRL was higher in more arid populations, with the lowest SRL in intermediate populations (aridity index *P* < 0.0001; Fig. 4c). Additionally, plants in the low watering regime had higher SRL than the high watering regime (watering regime *P* = 0.050; Fig. 4c), an effect that was strongest in the populations from arid areas (aridity index × watering regime *P* = 0.023; Fig. 4c).

*Avoidance:* populations from the center of the range had the greatest RMF, with lower RMF in populations from more arid areas (aridity index *P*  < 0.0001; Fig. 4d, see Table 2 for summary statistics). RMF displayed plasticity as plants in the low watering regime had greater RMF than in the high watering regime (watering regime *P* = 0.0005; Fig. 4d). Populations from more arid areas tended to have more dissected leaves (aridity index *P* = 0.017; Fig. 4e), and plants in the low watering regime also had more dissected leaves than the high watering regime (watering regime *P* = 0.046; Fig. 4e).

*Tolerance:* we did not find clinal variation in TLP (aridity index *P* = 0.077), however, TLP was reduced in the low watering treatment compared with the high watering treatment (watering regime *P* = 0.0001; Fig. 4f; see Table 2 for summary statistics). Plants from the center range survived the longest in the terminal drought (aridity index *P* = 0.0004; Fig. 4g). Additionally, plants in the high watering regime before terminal drought survived longer without water than plants in the low watering regime (watering regime *P* < 0.0001; Fig. 4g).

## Discussion

**Fig. 5:**
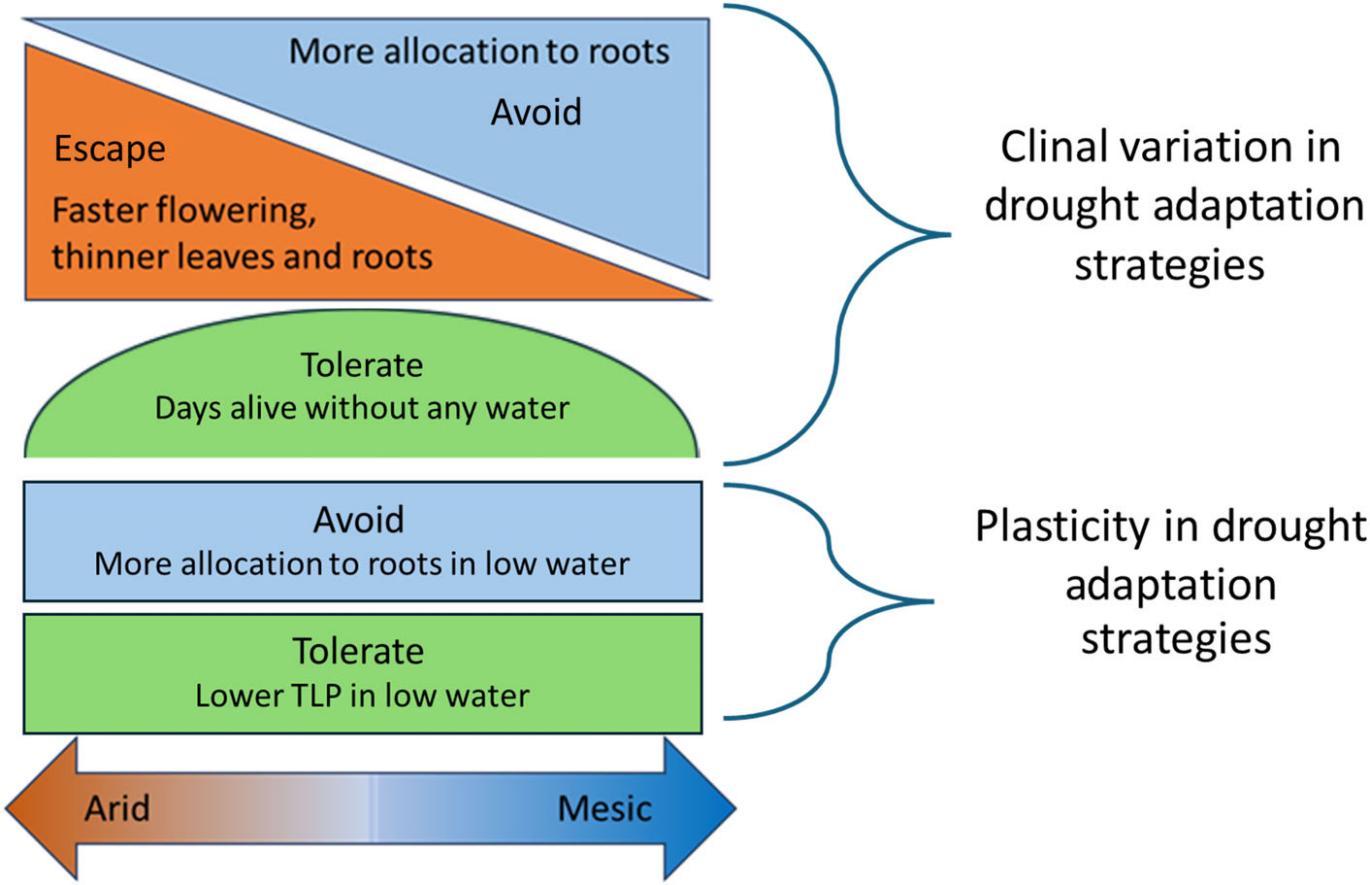
Conceptual diagram summarizing main findings between trait phenotypes and associated drought adaptation strategies of *Eschscholzia californica*. The shapes indicate clinal relationships in trait phenotypes, where triangles represent directional shifts, and the semi‐circle indicates the center of the range had the highest trait values for an associated drought adaptation strategy. The rectangles demonstrating plasticity are for traits that did not demonstrate detectable clinal variation in plasticity. Color indicates drought adaptation strategy, where orange is escape associated phenotypes, blue is avoidance associated phenotypes, and green is tolerance associated phenotypes for a given trait. Importantly, all populations experience an extended summer dry season, meaning that all populations are expected to possess drought adaptation strategies.

We found evidence for clinal variation in drought adaptation strategies across the distributional range of California poppy; populations from more arid areas exhibited trait phenotypes associated with escape strategies, while populations from intermediate aridity and more mesic areas had trait phenotypes associated with drought avoidance. The multivariate trait space occupied by each population generally trended toward resource acquisitive traits in populations from arid areas (i.e. high SLA, high SRL, and less negative TLP) and toward phenotypes associated with drought avoidance in populations from more mesic areas (i.e. high RMF, lower TLP, and longer survival in terminal drought). We found trait plasticity in response to low watering, with longer and thinner roots, greater investment in roots, more dissected leaves, and greater drought tolerance (i.e. lower TLP) in the low watering regime. SRL is the only trait to demonstrate clinal variation in plasticity, thus plasticity in traits associated with drought avoidance and tolerance were not constrained by the dominant drought adaptation strategy present in the population. We found that populations from sites with intermediate aridity survived the longest in the terminal drought and experienced the least water stress (i.e. highest predawn water potential; Fig. 5). Importantly, the clinal variation we observed in these traits suggests that drought adaptation strategies should not be viewed as categorical bins, but rather in terms of continuous traits that can co‐vary and depend critically on environmental context. These results are placed in a wider context in the paragraphs that follow.

The shift in drought adaptation strategies across the range of *E. californica* may be associated with variation in life history among populations (Brown, 2025). Populations on the northern, mesic end of the range are perennial and persist in a dormant state through dry summers by investing in roots to access deep soil moisture and store carbon for subsequent growing seasons (Boucher, 1985). By contrast, the southern, arid end of the species range experiences high interannual variation in rainfall (Dettinger *et al*., 2011), and these populations can display an annual life history favoring high reproductive allocation and low allocation to roots (Ryan & Cleland, 2021). The drought escape response may be limited to annual populations, and hence differences in life history within this species could limit the generality of our findings. However, there are other model species that similarly display life‐history variation across their range, with annual and perennial populations (e.g. *Mimulus guttatus*, Kooyers *et al*., 2015), and species where individuals can be either annual or perennial depending on environmental conditions and allocation of meristems (e.g. *Streptanthus tortuosus*, Gremer *et al*., 2020 and other examples reviewed by Friedman, 2020). Hence, the results of this study are likely generalizable beyond *E. californica*, especially because many species display variation in vital rates across their ranges (Doak & Morris, 2010; Merow *et al*., 2017; Sheth & Angert, 2018), and vital rates underlie both demography and life‐history strategies (Laughlin, 2023).

Some, but not all, traits investigated in this study mirrored expectations based on interspecific patterns across aridity gradients. For instance, we found that populations from more arid parts of the species range had thinner, less dense leaves (i.e. higher SLA) than the center‐range and mesic populations. Although this pattern is consistent with selection for a drought escape strategy in arid populations, it is opposite to the interspecific patterns observed for species turnover across moisture gradients (Cornwell & Ackerly, 2009). The pattern in SLA may also be driven by life history, as the populations that display annual life‐history strategies are in the arid portion of the range. Nevertheless, while intraspecific trait variation across environmental gradients is often hypothesized to be similar to interspecific patterns along the same gradient (Ackerly & Cornwell, 2007), the relationship is often found to be weaker within species than across species (Cornwell & Ackerly, 2009), or idiosyncratic across species (Fajardo & Siefert, 2018; Dong *et al*., 2020; Anderegg, 2023).

The differences between inter‐ and intraspecific patterns of trait variation along gradients of aridity could be further complicated by nonclimatic agents of local adaptation (Wadgymar *et al*., 2022). For instance, species adapted to arid regions often have high belowground allocation to access deep soil moisture (Yang *et al*., 2024); however, we found the opposite pattern. High allocation belowground can also be associated with greater competition from neighbors in productive environments (Rehling *et al*., 2021). For instance, Rowe & Leger (2011) found that populations of the native perennial bunchgrass *Elymus multisetus* tended to have greater allocation to roots in populations where they co‐occurred with invasive annual grasses. Past work has noted that *E. californica* is particularly vulnerable to competition from co‐occurring plant species (Cook, 1965). Hence, the northern populations of *E. californica* may have greater root allocation in part because of a history of selection in a more mesic, and potentially more competitive, context compared with southern populations.

Traits associated with the leaf economic spectrum often respond predictably to environmental stressors like aridity (Wright *et al*., 2004); however, other aspects of leaf morphology such as dissection may have less predictable associations with drought responses. Researchers have previously found that populations from arid sites had more dissected leaves than populations from mesic sites, mirroring interspecific patterns where species found in drier environments can have more highly dissected or lobed leaves (Kaproth *et al*., 2023, but see Nicotra *et al*., 2011). Leaf dissection may reduce leaf temperature, thereby retaining more water and enhancing avoidance responses (Kooyers *et al*., 2015); however, leaf temperature differences may not be realized in field settings (Leigh *et al*., 2017). Also, in our multivariate assessment, leaf dissection also appears to be orthogonal to other traits. In our study, although aridity index was a marginally nonsignificant predictor of leaf dissection, population‐level differences (included as a random term in our models) explained more variance in this aspect of leaf morphology than the fixed effect of site aridity. Importantly, there are several factors influencing leaf dissection, such as herbivory, that are nonmutually exclusive and may be driving the variability in leaf dissection findings (Nicotra *et al*., 2011; Wang *et al*., 2022).

Plant strategies in response to stressors like drought are associated with multiple complex traits often underlain by fundamental trade‐offs that define variation among species, but it is unclear whether trade‐offs constrain population‐level variation within species. In a study on interspecific trait coordination, Funk *et al*. (2024) found perennial species had greater belowground allocation and longer survival under terminal drought compared with annual species, consistent with a trade‐off between escape and avoidance. Among populations of *E. californica* we found negative correlations in avoidance and escape phenotypes but consistent plasticity in tolerance phenotypes. Our findings of a trade‐off between avoidance and escape strategies across populations is similar to the findings of a study among annual populations of *Mimulus guttatus* along an aridity gradient (Kooyers et al., 2015). While it is tempting to conclude that drought escape and avoidance are mutually exclusive strategies based on fundamental trade‐offs mediated by allocation, Welles & Funk (2021) found that populations of the invasive California wild radish from more arid sites had both greater belowground allocation and faster phenology, indicating selection for both escape and avoidance strategies simultaneously. The coordination between traits can be viewed from a network perspective on plant functional traits, where the allocation for growth in stems and leaf morphologies are central components of trait coordination (He *et al*., 2020). From this perspective, the unique and highly plastic leaf morphology of California poppy and California wild radish may contribute to these species' ability to coordinate between strategies. Additionally, trait associations may be greater in arid climates than temperate climates for herbaceous plants (Flores‐Moreno *et al*., 2019), suggesting that aridity may select for greater coordination in traits (Neves *et al*., 2021). Further work such as controlled breeding experiments to estimate heritability, phenotypic selection, or artificial selection studies would be required to evaluate whether selection on drought escape and avoidance traits is genetically constrained.

Trait plasticity is another key component of drought responses; we found plasticity in RMF and TLP, which were not constrained by clinal variation, yet plasticity in SRL did vary clinally (Fig. 5). Previous research has indicated that root traits are more plastic than leaf traits (Lozano *et al*., 2020; Dawson *et al*., 2024), which is also consistent with our findings that mainly root‐based traits had plasticity and clinal variation in plasticity. SRL is the only investigated trait with clinal variation in plasticity, consistent with other studies along water availability gradients (Münzbergová *et al*., 2017; Krushelnycky *et al*., 2020). However, the restricted variation in SRL for more mesic sites may be conflated with life history in our model system, whereas cold adaptations may have incurred constraints on plasticity in other studies (Münzbergová *et al*., 2017). We did not detect plasticity in SLA, which is in contrast to previous studies (Jiang *et al*., 2021) that could again be due to life history or alternatively due to light limitations from performing this experiment in a glasshouse that potentially constrained the plasticity of SLA in relation to water availability due to reduced light. We found reduced TLP in our low watering treatments, consistent with a global meta‐analysis (Bartlett *et al*., 2014) and expectations that lower TLP yields greater survivorship in drought for woody species (Bartlett *et al*., 2012). However, higher TLP may be positively associated with drought survival in herbaceous species (Sun *et al*., 2020), highlighting the need for more investigations into nonwoody drought adaptations and strategies.

The patterns of clinal variation and plasticity in response to low water stress described here have important implications for restoration, because wildflowers in the California floristic province are increasingly threatened by drought (Harrison *et al*., 2015). The prevailing wisdom for seed sourcing in restoration projects is to focus on acquiring seeds from the closest possible source; however, that wisdom has been questioned in the face of climate change (Havens *et al*., 2015; Nolan *et al*., 2023). Instead, introducing seeds from populations already experiencing warmer and drier conditions could introduce adaptive alleles and accelerate adaptation to climate change (i.e. assisted gene flow, Aitken & Whitlock, 2013). Indeed, experimental crosses have demonstrated the potential for improving the survivorship and performance of populations vulnerable to climate change (Sexton *et al*., 2011; Bontrager & Angert, 2019). A number of common garden studies have found that populations from arid areas outperform populations from mesic areas even under high water conditions (Pratt & Mooney, 2013; Barton *et al*., 2020; Ramírez‐Valiente *et al*., 2021, 2022). This is in contrast to the expected trade‐off between adaptation to drier microclimates and competitive ability under benign conditions (Gade & Metz, 2024), where the increased performance of arid adapted populations in high water conditions could indicate populations in currently mesic areas are already outside of their climatic optima due to climate change in recent decades. Looking forward, it will be important to understand whether species of conservation concern often exhibit clinal variation, in which case assisted gene flow may be a viable strategy for accelerating adaptive contemporary evolution to changes in climate (Aitken & Whitlock, 2013). However, in the absence of adaptive clinal variation, selective breeding within provenances would likely be a more successful strategy for enhancing climate adaptation (Candido‐Ribeiro & Aitken, 2024).

There are caveats that are important to the interpretation of our results. First, we used wild‐collected seeds without a refresher generation in this experiment. While the use of wild‐collected seeds is commonplace in experiments seeking to understand clinal variation or local adaptation (e.g. Hereford, 2009, with other examples reviewed by Baughman *et al*., 2018), the phenotype expressed will be the product of both the source genotype and maternal effects associated with the environmental conditions experienced by the parental generation. This can be seen as a limitation, but it can also be seen as a benefit, because wild‐collected seeds result in a ‘realized phenotype’ in the experiment that most realistically replicates the phenotypic response of the focal population to the experimental treatments (Auge *et al*., 2023). Additionally, we used biomass as a proxy for plant fitness rather than measuring reproductive output and seed viability due to low flowering and pod production in glasshouse conditions for this species (Ryan & Cleland, 2021); however, biomass estimates are often highly associated with plant fitness (Younginger *et al*., 2017). Pot experiments incur additional caveats. For instance, the benefits of a drought avoidance strategy may be muted because increased root allocation does not allow the plant to access deep soil layers with increased soil moisture. Furthermore, experimental findings are always limited to the unique set of conditions that were imposed; it is possible for instance, that mesic populations could have experienced water stress even at our high level of watering, because the glasshouse was located in the southern part of the range. We think this is unlikely, however, because we did not see clinal variation in TLP, suggesting consistent hydraulic tolerances across the populations.

In conclusion, our results show trade‐offs in drought strategies across the native range of an iconic herbaceous wildflower. In this case, frameworks designed to describe suites of traits that contribute to variation in plant strategies across species (Ludlow & Muchow, 1990; Kooyers, 2015) largely predicted the manner that traits co‐varied within a species across an environmental gradient (aridity). Furthermore, *E. californica* displayed plasticity in traits associated with drought avoidance and tolerance, and this plasticity was not constrained by the population's dominant drought response strategy. By contrast, SRL displayed clinal variation in plasticity that mirrored the overall clinal variation, with thinner roots in both the arid end of the distribution range and in response to drought stress. These results have implications for restoration practitioners considering assisted gene flow as a mechanism to accelerate adaptation to increasingly arid conditions predicted for future decades.

## Competing interests

None declared.

## Author contributions

STS and EEC designed the study. JK, JPS, RB and EEC collected seeds. STS, KL and FT conducted the study. EEC, JPS and CM received funding for the study. STS and EEC wrote the manuscript, with contributions and edits from all co‐authors.

## Disclaimer

The New Phytologist Foundation remains neutral with regard to jurisdictional claims in maps and in any institutional affiliations.

## Supporting information


**Dataset S1** Data collected and analyzed for this experiment.

**Fig. S1** Covariance matrix of environmental variables.
**Fig. S2** Flowering rates of *Eschscholzia californica* populations.
**Fig. S3** Relationships between days from emergence to flower with population probability of flowering, specific root length, and specific leaf area for *Eschscholzia californica*.
**Fig. S4** Transformed values of volumetric water content, shoot mass, and predawn water potential of *Eschscholzia californica* populations.
**Fig. S5** Transformed values of specific leaf area, specific root length, root mass fraction, leaf dissection index, turgor loss point, and days alive in terminal drought for *Eschschozlia californica*.
**Table S1** Average values for environmental variables of home site of Eschscholzia californica populations investigated.
**Table S2** Count of Eschscholzia californica maternal lines that successfully emerged within each population and sum of replicates for each population.
**Table S3** Model selection table comparing linear and quadratic regressions of *Eschscholzia californica* trait responses.
Please note: Wiley is not responsible for the content or functionality of any Supporting Information supplied by the authors. Any queries (other than missing material) should be directed to the *New Phytologist* Central Office.

## Data Availability

The data that support the findings of this study are available in the Supporting Information of this article (Dataset S1).
