# Supplementary material for: Can species adapt to drought using multiple strategies? Lessons from the California poppy
Source: New Phytol. 2026 Apr 2;250(5):2918–32. doi: 10.1111/nph.71105 (PMC13150309; doi:10.1111/nph.71105)
Supplement: Supplementary file 2 — Fig. S1 Covariance matrix of environmental variables. Fig. S2 Flowering rates of Eschscholzia californica populations. Fig. S3 Relationships between days from emergence to flower with population probability of flowering, specific root length, and specific leaf area for Eschscholzia californica. Fig. S4 Transformed values of volumetric water content, shoot mass, and predawn water potential of Eschscholzia californica populations. Fig. S5 Transformed values of specific leaf area, specific root length, root mass fraction, leaf dissection index, turgor loss point, and days alive in terminal drought for Eschschozlia californica. Table S1 Average values for environmental variables of home site of Eschscholzia californica populations investigated. Table S2 Count of Eschscholzia californica maternal lines that successfully emerged within each population and sum of replicates for each population. Table S3 Model selection table comparing linear and quadratic regressions of Eschscholzia californica trait responses. Please note: Wiley is not responsible for the content or functionality of any Supporting Information supplied by the authors. Any queries (other than missing material) should be directed to the New Phytologist Central Office. [file NPH-250-2918-s002.pdf]

New Phytologist Supporting Information

Article title: Can species adapt to drought using multiple strategies? Lessons from the California poppy

Authors: Stuart T Schwab, Kristal Lam, Finn Thornton, Rachel Brown, Joe Kesler, Cory Merow, Jason P. Sexton, Elsa E. Cleland

Article acceptance date: 23 February 2026

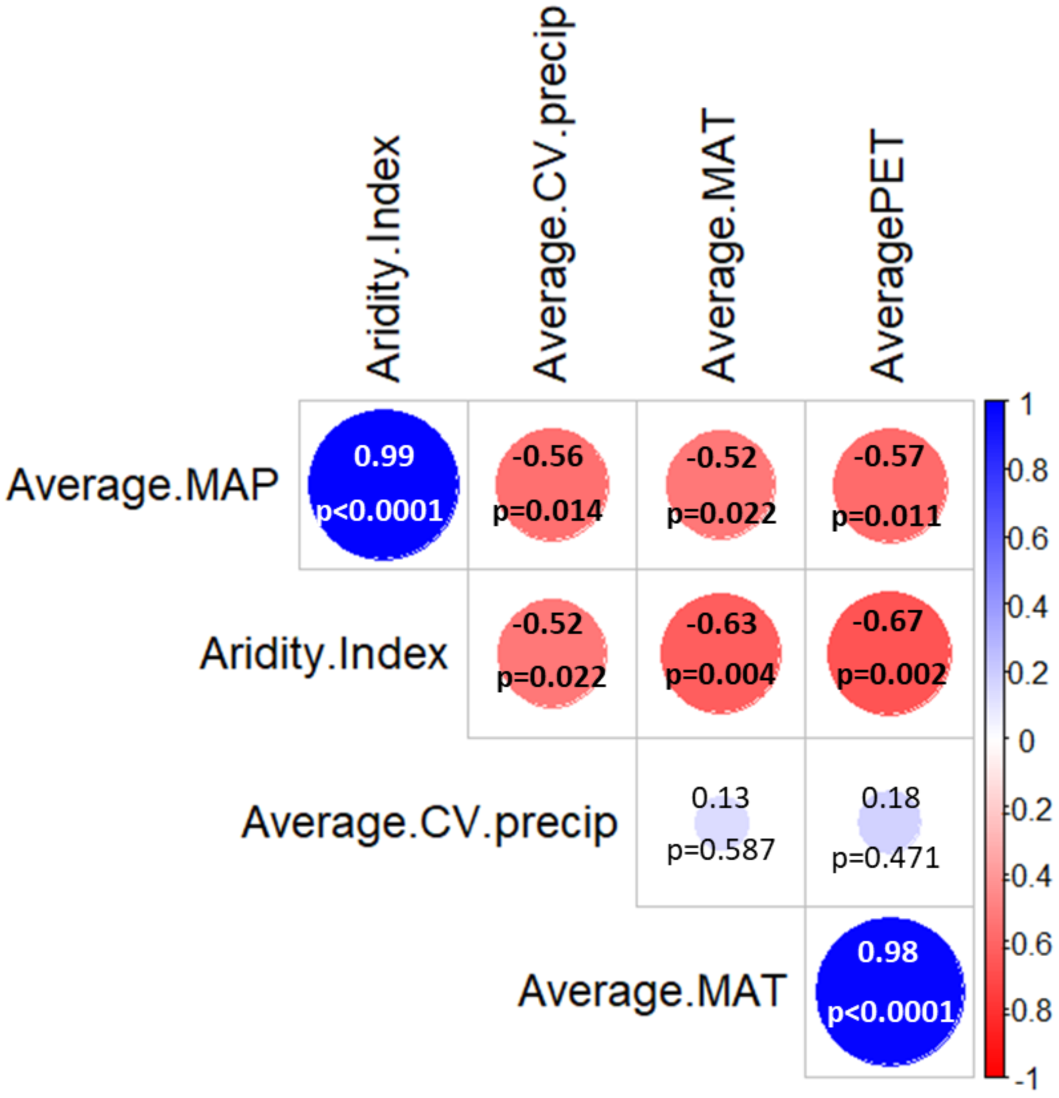

Supplemental Figure 1) Correlation matrix of considered environmental variables, downloaded from PRISM database and averaged over the past 30 years. Aridity index is calculated as (precipitation/potential evapotranspiration), average.cv.precip is the average coefficient of variation of precipitation (standard deviation/mean), Average MAT is the mean annual temperature, average PET is the potential evapotranspiration, and average MAP is the mean annual precipitation. The top number within each cell is the correlation coefficient, with the p value for each correlation listed below. Larger, darker, circles have stronger correlations where blue is positive and red is negative.

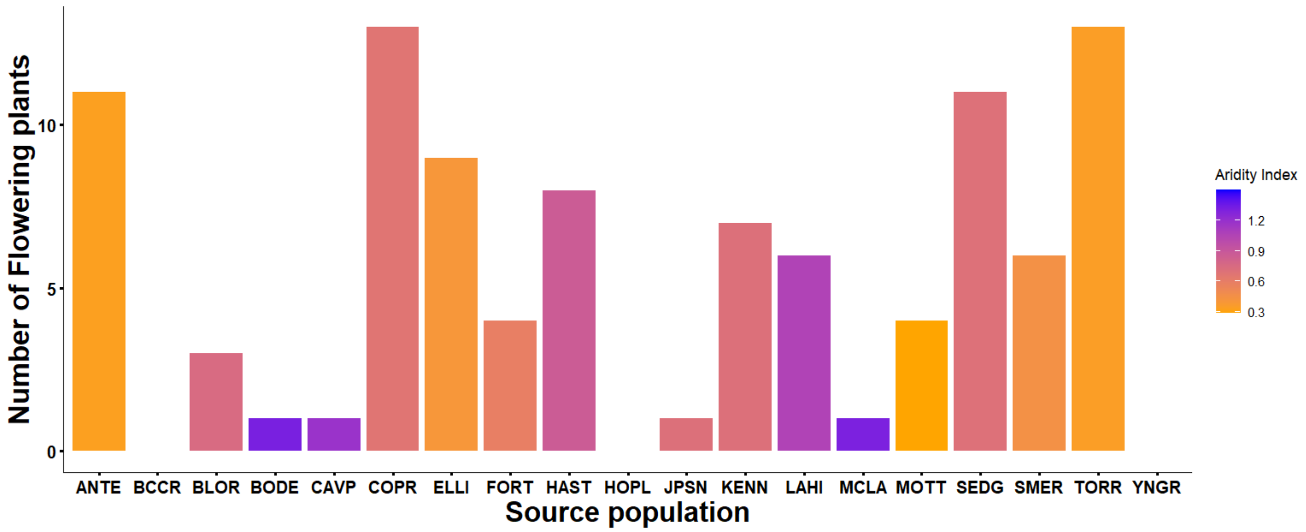

Supplemental Figure 2) Counts of flowering *Eschscholzia californica* plants for each population within the continued watering treatment. Color indicates aridity index, where lower values (in orange) are more arid sites, while higher values (purple) are more mesic sites. Three sites did not have any flowering individuals.

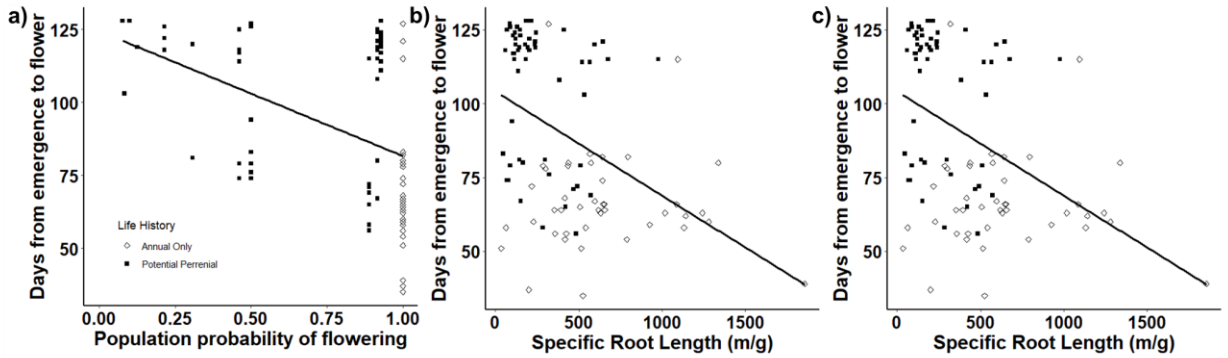

Supplemental Figure 3) Correlation between days from emergence to flower, population level probability of flowering, and resource acquisition traits of *Eschscholzia californica.* A) Population level probability of flowering relationship with days from emergence to flowering, with shape demonstrating life history. B) Specific Leaf Area relationship with days from emergence to flowering, with shape demonstrating life history. C) Specific Root Length relationship with days from emergence to flowering, with shape demonstrating life history

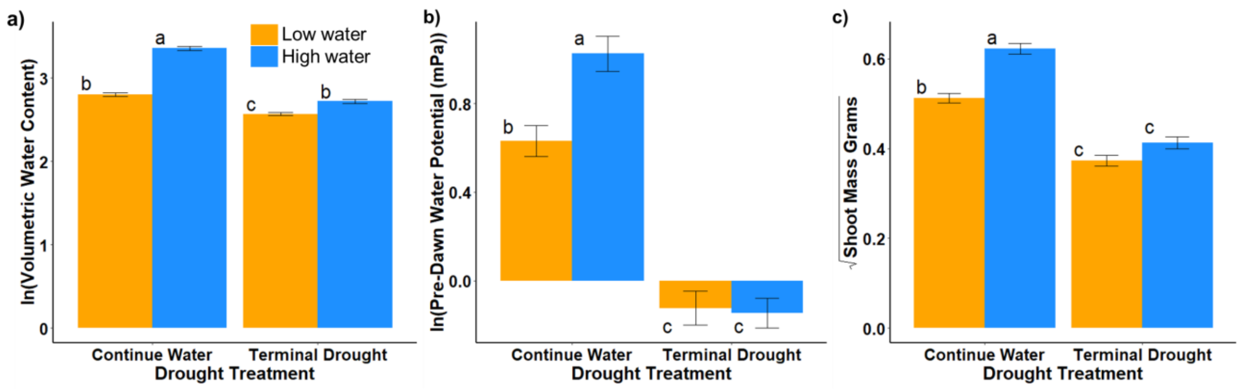

Supplemental Figure 4) Transformed values for volumetric water content (a), predawn water potential (b), and shoot mass (c) of *Eschscholzia californica* in response to watering and terminal drought treatments. Color indicates high or low watering (orange=low water, blue=high water) and bars represent standard error. Letters indicate results of Tukey honestly significant difference test. See Table 2 for full summary statistics.

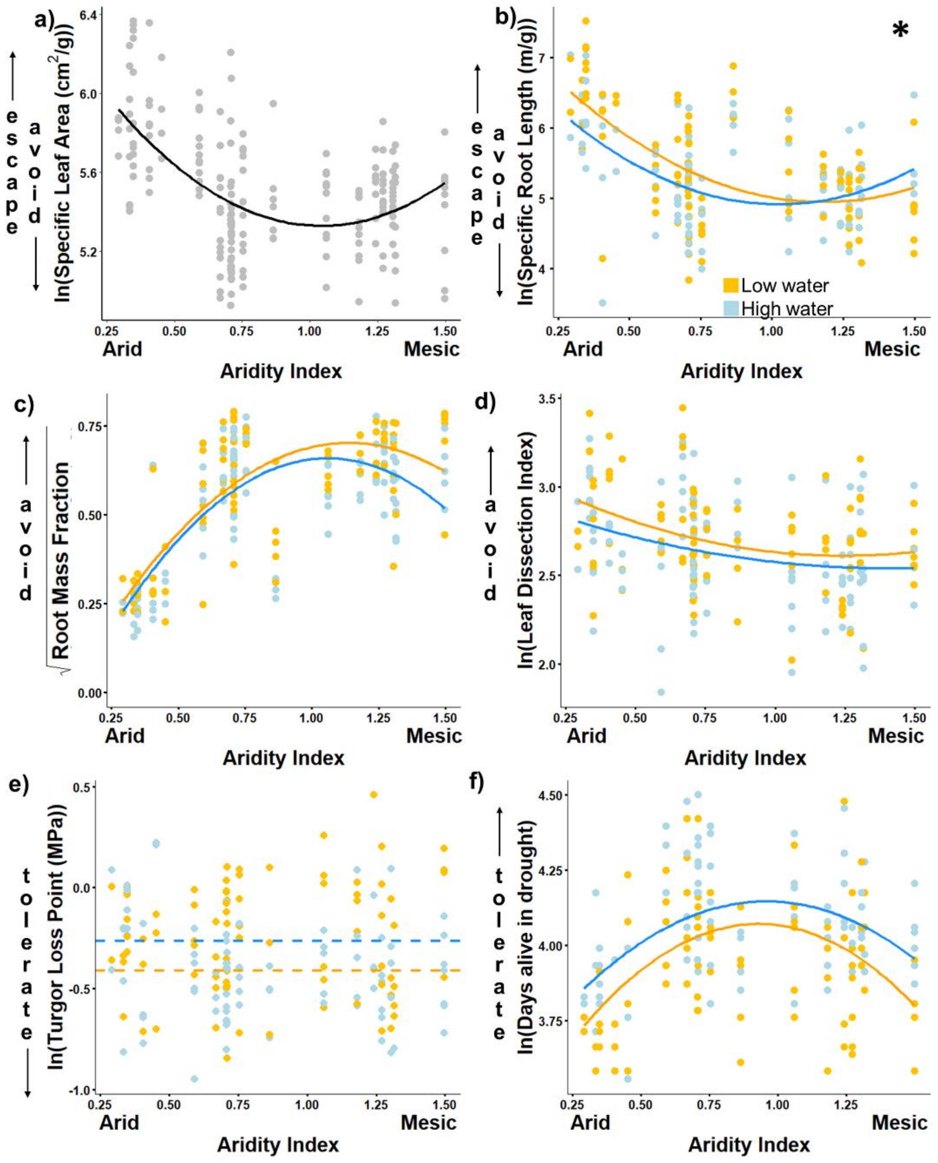

Supplemental Figure 5) Clinal variation in *Eschscholzia californica* functional trait phenotypes after data transformations for analysis. Summary statistics for displayed relationships are in Table 2. Color indicates different watering treatments; orange is low water, blue is high water, and black indicates there was no influence of watering treatment on the relationship. Dashed color lines indicate that clinal variation did not have a detectable impact, and watering regimes were significantly different (Table 2). A) Specific Leaf Area (SLA (cm^2/g)). B) Specific Root Length (SRL (m/g)). C) Root Mass Fraction D) Leaf dissection index (cm^{-1}). E) Turgor Loss Point (TLP, Mpa). F) Days survived in terminal drought treatment.

Supplemental Table 1) Average values for environmental variables of home site of *Eschscholzia californica* populations investigated. Data was downloaded from the PRISM database (PRISM group 2023) to calculate the 30 year norms. Average CV precip is the coefficient of variation in precipitation (mean/standard deviation). Average MAP is the mean annual precipitation, while average MAT is the mean annual temperature. Average PET is the potential evapotranspiration as calculated with the Thornthwaite function. Aridity index is (precipitation/PET).

Site abbreviated	Full Site Name	Longitude	Latitude	Average.CV.precip	Average.MAP	Average.MAT	AveragePET	Aridity.Index
ANTE	Antelope Valley State Poppy Reserve	-118.401	34.73214	155.7177	22.18997	17.00862	66.22222	0.335083
BCCR	Big Chico Creek Ecological Reserve	-121.068	38.97694	125.2488	76.58336	15.86437	60.27696	1.270525
BLOR	Blue Oak Ranch	-121.753	37.38972	135.2618	42.85787	14.89052	56.79993	0.754541
BODE	Bodega Bay	-123.068	38.31583	139.7312	66.7177	11.33132	50.7151	1.315539
CAVP	Canyon Valley Preserve Placer Land Trust	-121.052	38.93148	125.7799	71.7192	16.01207	60.73634	1.180828
COPR	Coil Oil Point Reserve	-119.88	34.41194	166.3533	38.46925	14.89454	57.46732	0.669411
ELLI	Elliot Reserve	-117.089	32.8928	140.9109	26.45431	17.59885	65.0909	0.406421
FORT	Fort Ord	-121.778	36.68611	140.5008	32.31086	13.5408	54.56925	0.592107
HAST	Hasting's Reserve	-121.555	36.38472	148.2639	48.55394	14.63506	56.13945	0.864881
HOPL	Hopland Research and Extension Center	-123.08	38.99523	137.3813	83.87086	14.53822	55.99783	1.497752
JPSN	Jepson Prairie	-121.824	38.27733	146.1071	42.38307	15.74397	59.77566	0.709036
KENN	Kenneth Norris Rancho Marino Reserve	-121.07	35.52861	157.0432	39.07851	13.70603	55.0875	0.70939
LAHI	Landel's Hill Big Creek	-121.588	36.05924	148.7819	58.18891	13.73793	54.86586	1.060567
MCLA	McLaughlin Reserve	-122.41	38.85934	144.1932	75.81661	15.17931	58.07125	1.305579
MOTT	Motte Rimrock Reserve	-117.257	33.79944	155.916	19.70463	17.83879	67.18054	0.293309
SEDG	Sedgewick Reserve	-120.054	34.70194	159.6301	43.71374	16.53506	61.80518	0.707283
SMER	Santa Margarita Ecological Reserve	-117.171	33.4583	149.4512	30.4502	18.1592	67.18592	0.453223
TORR	Torry Pines State Reserve	-117.258	32.92432	144.6875	21.83477	16.89253	62.64039	0.348573
YNGR	Younger Reserve	-122.067	36.94944	144.5166	66.32557	12.93103	53.40248	1.241994

Supplemental Table 2) Count of *Eschscholzia californica* maternal lines that successfully emerged within each population and sum of replicates for each population.

Source population location	Count of Maternal Line							Sum per population
	A	B	C	D	E	F	G	
Antelope Valley State Poppy Reserve	3	3	0	4	2	2	2	16
Big Chico Creek Ecological Reserve	4	4	4	4	4	4	4	28
Blue Oak Ranch	4	3	4	4	4	4	4	27
Bodega Bay	4	1	1	1	4	0	3	14
Canyon Valley Preserve Placer Land Trust	4	1	1	4	4	1	4	19
Coil Oil Point Reserve	4	4	4	4	4	4	4	28
Elliot Reserve	1	1	1	4	4	0	3	14
Fort Ord	4	4	4	4	2	3	3	24
Hasting's Reserve	4	0	0	4	4	3	4	19
Hopland Research and Extension Center	4	4	2	4	3	4	4	25
Jepson Prairie	4	4	1	4	1	4	4	22
Kenneth Norris Rancho Marino Reserve	4	4	4	4	4	4	4	28
Landel's Hill Big Creek	4	4	4	4	3	4	4	27
McLaughlin Reserve	4	4	4	4	4	4	1	25
Motte Rimrock Reserve	0	4	0	4	0	0	0	8
Sedgewick Reserve	4	4	4	4	4	2	0	22
Santa Margarita Ecological Reserve	3	0	1	0	4	3	3	14
Torry Pines State Reserve	3	4	4	2	4	4	4	25
Younger Reserve	4	3	4	1	0	4	4	20

Supplementary Table 3: Model selection table comparing linear and quadratic regressions of *Eschscholzia californica* trait responses, including transformed models, where needed, to normalize model residual distribution. The first number is the Akaike Index Criterion (AIC), followed by a Shapiro-wilks test for normality of the distribution of model residuals. Bolded models were chosen to use in the main analysis.

	**Linear untransformed**		**Linear Transformed**		**Quadratic untransformed**		**Quadratic transformed**	
	AIC	s-w test result	AIC	s-w test result	AIC	s-w test result	AIC	s-w test result
**VWC**	2390	<0.0001	-22	0.006	2350	<0.0001	**-40**	**0.005**
**Predawn wp**	839	<0.0001	838	0.0009	812	<0.0001	**816**	**0.002**
**shoot mass**	-494	<0.0001	-533	0.34	-508	<0.0001	**-543**	**0.297**
**Probability Flowering**	**160**	**<0.0001**	NA (binomial)		163	<0.0001	NA (binomial)	
**SLA**	2281	<0.0001	58	0.092	2239	<0.0001	**38**	**0.028**
**SRL**	2665	<0.0001	372	0.276	2621	<0.0001	**357**	**0.238**
**RMF**	-284	0.002	-318	0.0004	-296	0.003	**-334**	**0.0006**
**dissection index**	1189	0.009	73	0.809	1172	0.009	**68**	**0.846**
**TLP**	152	0.248	39	0.258	146	0.189	**33**	**0.384**
**days alive no water**	1442	<0.0001	-101	0.072	1410	<0.0001	**-118**	**0.059**
